# Endobronchial ultrasound–guided transbronchial tunnel Cryobiopsy for mediastinal lymphadenopathy (with video)

**DOI:** 10.1097/eus.0000000000000063

**Published:** 2024-07-16

**Authors:** Mingming Deng, Run Tong, Ziwen Zheng, Gang Hou

**Affiliations:** National Center for Respiratory Medicine; State Key Laboratory of Respiratory Health and Multimorbidity; National Clinical Research Center for Respiratory Diseases; Institute of Respiratory Medicine, Chinese Academy of Medical Sciences; Department of Pulmonary and Critical Care Medicine, Center of Respiratory Medicine, China-Japan Friendship Hospital, Beijing, China.

Endobronchial ultrasound–guided transbronchial cryobiopsy (EBUS-TBCB) has gained widespread attention as a diagnostic technique for patients with unknown mediastinal lymphadenopathy.^[1,2]^ EBUS-TBCB requires a high-frequency needle knife to incise the airway for the cryoprobe to be inserted into the lymph node.^[3,4]^ Bronchoscopic electrocautery is a therapeutic technique with a high requirement for bronchoscopists, which may limit its application. Moreover, the potential risks to the airways after electrocautery remain unclear. Hence, we have developed endobronchial ultrasound–guided transbronchial tunnel cryobiopsy (EBUS-TTCB) using the disposable transbronchial puncture dilation catheter (BroncTru™ AK-91-55; Broncus®, Hangzhou, China) [Figure 1]. Herein, we report a case of successful EBUS-TTCB.

**Figure 1:**
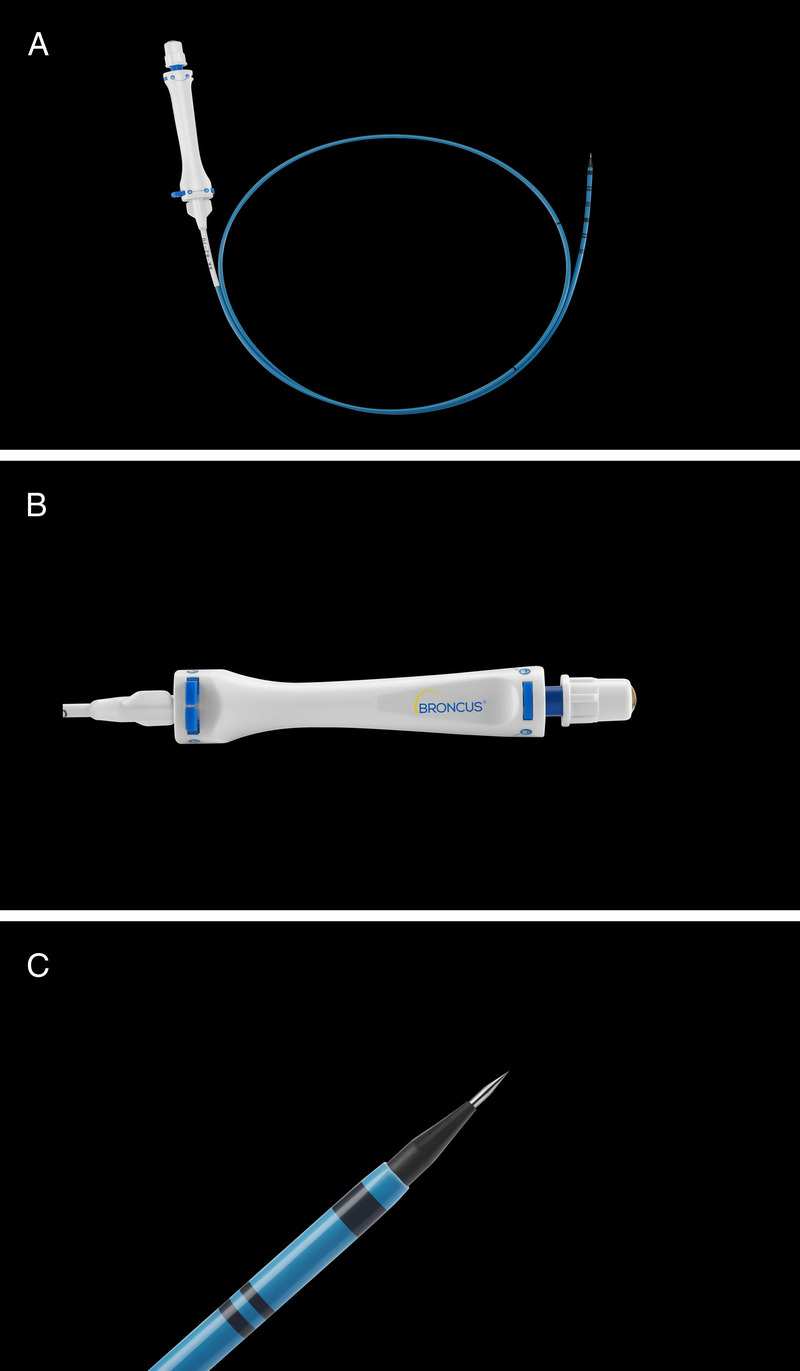
Images of the disposable transbronchial puncture dilation catheter. The disposable transbronchial puncture dilation catheter (BroncTru™; Broncus, Hangzhou, China) consists of a core needle and a sheath. (A) The overview of the disposable transbronchial puncture dilation catheter. (B) The handle of the disposable transbronchial puncture dilation catheter. (C) The tip of the disposable transbronchial puncture dilation catheter.

A 50-year-old man whose chest computed tomography revealed diffuse ground-glass opacities in both lungs and multiple mediastinal lymphadenopathies [Figure 2] underwent EBUS-TTCB [Figure 3, and Supplement Video]. An ultrasonic bronchoscope (Olympus UC260F, Tokyo, Japan) was inserted into the trachea through a laryngeal mask under moderate sedation and topical anesthesia of the upper airway. The station 7 lymph node was enlarged, the size and bloody supply of which were assessed to ensure the safety of TTCB. A BroncTru™ catheter was inserted into the working channel of the ultrasonic bronchoscope. When the tip of the catheter spiked the airway wall, the catheter bluntly expanded the puncture point and arrived at the target lymph node under EBUS guidance. The core needle was withdrawn, and a sheath was left as a tunnel between the airway wall and target lymph node. The 1.1-mm cryoprobe (Erbe 20402-401, Tübingen, Germany) entered the target lymph node through the tunnel, which could be directly monitored, and the distance between the tip of the cryoprobe and the border of station 7 lymph node was measured using EBUS. After confirming that the distance was >5 mm, the probe was cooled with liquid carbon dioxide for 9 seconds. The probe was then fixed to the ultrasound bronchoscope as one piece with the left hand and pulled out to complete the EBUS-TTCB. EBUS-TTCB was performed 3 times [Figure 4]. No active bleeding, pneumothorax, or pneumomediastinum was observed after TTCB. A definitive diagnosis of pneumoconiosis was established [Figure 5].

**Figure 2:**
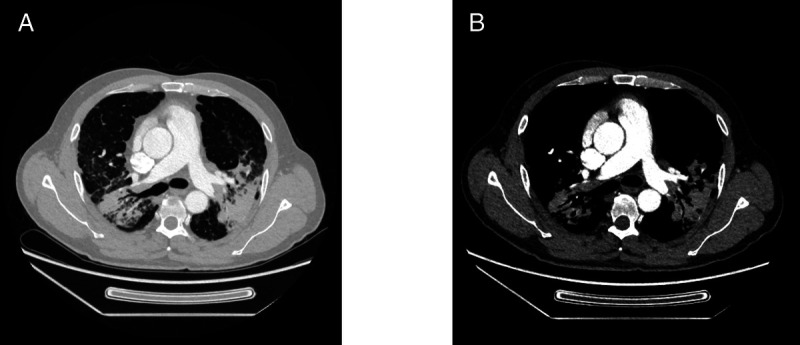
Chest CT findings. The chest CT of the patient reveals diffuse ground-glass opacities in both lungs, and multiple mediastinal lymphadenopathies. (A) CT scan in lung window. (B) CT scan in mediastinal window. CT: Computed tomography.

**Figure 3:**
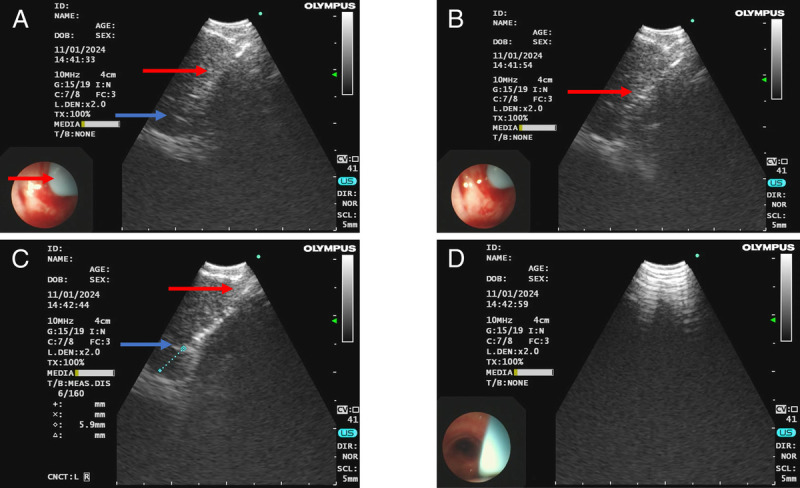
Flowchart for the EBUS-TTCB procedure. (A) The catheter bluntly expands the puncture point and reaches the target lymph node under the guidance of endobronchial ultrasound. The red arrow shows the sheath, and the blue arrow shows the tip of the catheter. (B) After withdrawing the core needle of the catheter, the catheter sheath is left as a tunnel between the airway and target lesion. The red arrow shows the tip of the catheter. (C) The distance between the tip of cryoprobe and target lesion is ensured to be greater than 5 mm. The red arrow shows the sheath of the catheter, and the blue arrow shows the tip of cryoprobe. (D) The probe and tissues are removed with the sheath. EBUS-TTCB: Endobronchial ultrasound–guided transbronchial tunnel cryobiopsy.

**Video Legend** Videos are only available at the official website of the journal (http://www.eusjournal.com).

**Figure 4:**
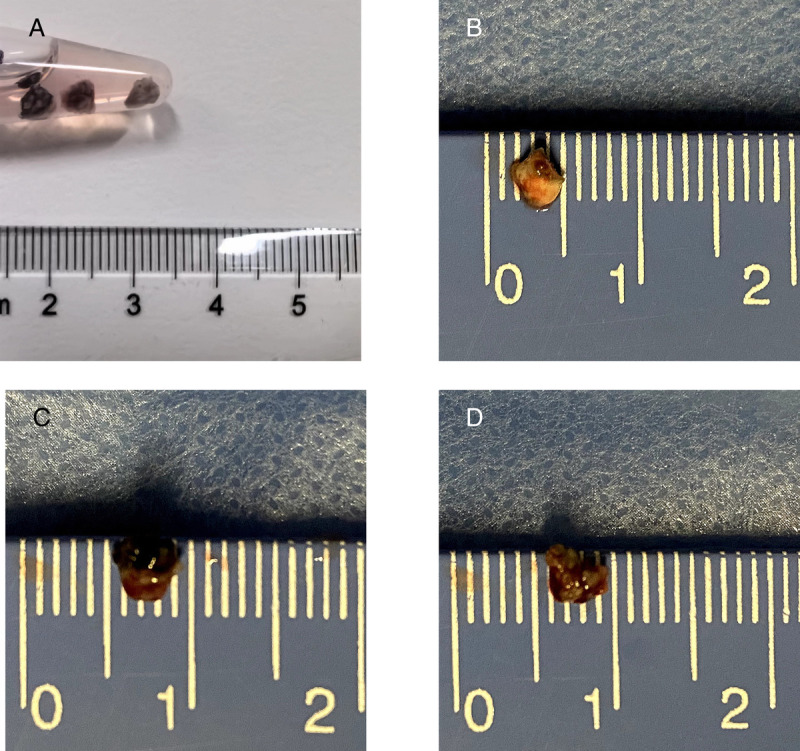
Samples obtained using EBUS-TTCB, with a mean diameter of 5 mm, without any airway tissues attached to them. (A) The overview of the 3 samples obtained by EBUS-TTCB. (B) Sample obtained by the first cryobiopsy (size, 4 mm × 4 mm). (C) Sample obtained by the second cryobiopsy (size, 5 mm × 4 mm). (D) Sample obtained by the third cryobiopsy (size, 5 mm × 4 mm). EBUS-TTCB: Endobronchial ultrasound–guided transbronchial tunnel cryobiopsy.

**Figure 5:**
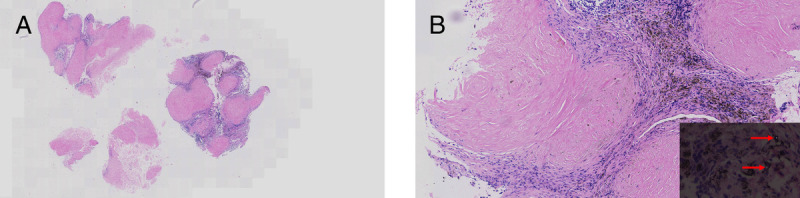
Histopathological evaluation of the sample. (A) Pathological section analysis of the sample (HE, 40×). (B) Pathological section analysis of the sample (HE, 100×); birefringent particles were seen under a polarized light microscope (red arrow). EBUS-TTCB: Endobronchial ultrasound–guided transbronchial tunnel cryobiopsy; HE: Hematoxylin-eosin staining.

EBUS-TTCB may be an easier and feasible procedure for sampling in mediastinal lymphadenopathy.

## Declaration of Patient Consent

The authors certify that they have obtained all appropriate patient consent forms. In the form, the patient has given his consent for his images and other clinical information to be reported in the journal. The patient understands that his name and initials will not be published and due efforts will be made to conceal his identity, but anonymity cannot be guaranteed.
